# Cancer, collapse, and the politics of somatic evolution

**DOI:** 10.1093/emph/eoag004

**Published:** 2026-01-29

**Authors:** Michel Salzet

**Affiliations:** Univ. Lille, Inserm, CHU Lille, U1192 -Protéomique Réponse Inflammatoire Spectrométrie de Masse (PRISM), F-59000 Lille, France; Institut Universitaire de France, Ministère de l’Enseignement Supérieur, de la Recherche et de l’Innovation, 1 rue Descartes, 75231 PARIS CEDEX 05, France; Equipe Labellisée Ligue Contre le Cancer, Paris, France

**Keywords:** cancer evolution, multicellularity, cooperation and cheating, tumour ecology, phenotypic plasticity, adaptive therapy

## Abstract

Multicellularity rests on a social contract: cells give up autonomy in exchange for shared resources, division of labour, and protection. Cancer is what happens when that contract collapses, and somatic evolution runs loose inside the body. Here, the multicellular social contract is used as a framework to recast tumour suppressors, tissue architecture, and immune surveillance as enforcement devices, and cancer as a ‘rogue society’ where cheater lineages exploit public goods, remodel niches, and build hierarchies. This perspective links clonal evolution, phenotypic plasticity, and therapy resistance to the ‘politics’ of rules and enforcement, and points towards therapies that do not just kill malignant cells but actively reshape the conditions under which somatic evolution proceeds.

## INTRODUCTION

Classical evolutionary oncology describes tumours as evolving cell populations shaped by mutation, selection, and drift within tissue ecosystems [1–4]. This view has profoundly influenced how intratumour heterogeneity, metastasis, and therapy resistance are understood. Yet most of this work is framed in the language of genetics and ecology, such as drivers and passengers, fitness landscapes, niches, and selective sweeps. A complementary perspective starts one step earlier, at the major transition to multicellularity [5]. Multicellular organisms persist only because vast numbers of cells cooperate. They divide when asked, differentiate on cue, share resources, and accept programmed death for the benefit of the whole [6]. Cooperation is therefore an evolvable strategy rather than a default state [7]. In some lineages, unicellular and multicellular phases alternate depending on ecological conditions [8–10]. This arrangement is not automatic. It must be stabilized against cheaters, cells that proliferate too much, ignore differentiation cues, or hoard resources [11–13]. The same principles of mutation, heritable variation, and selection that enabled complex multicellularity also allow its erosion when ecological and selective conditions favour defection. From the cancer cell’s perspective, apparently ‘chaotic’ tumour behaviours can be adaptive. Selection favours traits that relax constraints, remodel microenvironments, and open new evolutionary opportunities. Recent evolutionary reinterpretations of the hallmarks of cancer frame hallmark traits as eco-evolutionary processes that emerge during progressive tumour evolution [14, 15]. Evolution has therefore built what we might call a multicellular social contract. An implicit set of rules and enforcement mechanisms that coordinate cell behaviour and penalize defectors. This contract has three broad pillars. First, intrinsic policing via tumour suppressors and damage checkpoints constrains selfish proliferation. Second, extrinsic policing via immune surveillance and tissue architecture removes or isolates deviant cells. Third, developmental and metabolic constraints structure who can do what, where, and when, limiting opportunities for cheating [16–18]. When these pillars are strong, somatic evolution is constrained within a narrow corridor of allowed behaviours. When they are weakened or bypassed, cancer can be understood as a failure of contract enforcement. In what follows, the social-contract metaphor is used to organize familiar pieces of evolutionary oncology into four themes: (i) reinforcement and erosion of multicellular order, (ii) tumour public-goods dynamics and free-riding, (iii) plasticity and hierarchy as political strategies of resistance, and (iv) therapeutic attempts to renegotiate or exploit the contract.

Analytical status of the framework: the multicellular social-contract framework is not proposed as a formal evolutionary model, nor does it attribute intentions to cells. The phrase ‘social contract’ is borrowed from political philosophy [19, 20] but is used here as shorthand for the constraints and enforcement mechanisms that stabilize somatic cooperation. It is used here as a heuristic lens that organizes enforcement mechanisms, rule-breaking, and adaptive responses across scales, and that complements existing ecological metaphors (e.g. tumours as ecosystems or arms races) by keeping attention on the constraints that stabilize cooperation (checkpoints, tissue architecture, immune surveillance) and on how tumours systematically erode or bypass them [21].

## MULTICELLULAR ORDER AND ITS BREAKDOWN

### Building and enforcing the multicellular contract

**Figure 1:**
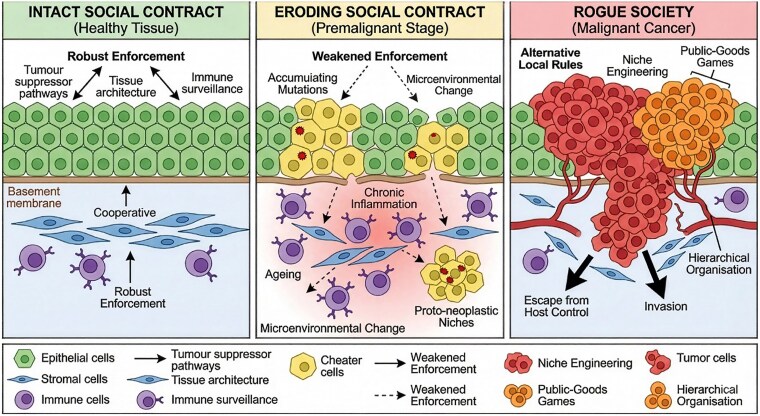
Conceptual model of carcinogenesis as a progressive breakdown of the multicellular ‘social contract.’ **Left (intact social contract, healthy tissue):** Cooperative epithelial cells (green) are constrained by robust enforcement mechanisms, tumour-suppressor pathways, preserved tissue architecture, and immune surveillance (purple), maintaining homeostasis above an intact basement membrane with supportive stroma (blue). **Middle (eroding social contract; premalignant stage):** Enforcement weakens as mutations accumulate, allowing the emergence and expansion of ‘cheater’ clones (yellow), concomitant microenvironmental shifts (e.g. ageing and chronic inflammation) further undermine control, fostering proto-neoplastic niches and patchy, unstable tissue organization (dashed/attenuated enforcement). **Right (rogue society; malignant cancer):** Tumour cells (red) establish alternative local rules, actively engineer niches and engage in public-goods dynamics, adopting hierarchical organization; these innovations enable escape from host control, invasion across tissue boundaries, and progressive malignant expansion.

Multicellularity requires that cells accept constraints on proliferation, movement, and identity. Tumour suppressors and checkpoints provide the internal legal code of this contract. Genes such as TP53, RB1, or APC integrate signals about DNA damage, nutrient status, and tissue context, enforcing cell-cycle arrest, senescence, or apoptosis when conditions are not met [22–24]. In contract language, they specify when a cell is allowed to replicate, and what penalties follow if rules are violated. Beyond individual genes, tissue architecture and stem-cell hierarchies act as structural enforcement. Stem cells occupy protected niches with tightly regulated division; their progeny differentiate and are eventually removed. This arrangement limits the number of long-lived, self-renewing cells that can accumulate dangerous mutations. Likewise, epithelial polarity, basement membranes, and stromal scaffolds constrain where cells can move and how they interact with neighbours ([94]; [25, 26]). Figure 1 summarizes these evolutionary reinforcements of the contract, such as intrinsic checkpoints, niche structures, differentiation hierarchies, and immune surveillance. Together, they reduce the effective population size of potentially malignant cells, curtail opportunities for clonal expansion, and make cheating costly. Thus, Intrinsic policing (tumour suppressors and checkpoints) constrains inappropriate proliferation; extrinsic policing (immune surveillance and tissue architecture) removes or contains deviant cells, and developmental/metabolic constraints (differentiation programmes, niche limits, and resource structure) reduce opportunities for cheating. Together, these mechanisms stabilize cooperation and limit somatic evolution. Comparative genomic and functional studies in elephants, bowhead whales, and naked mole-rats show lineage-specific mechanisms that reinforce the multicellular ‘contract’ and counteract cancer risk (Supplementary Fig. 1**,**  Supplementary Table 1) [27–30].

### Erosion of enforcement

Cancer begins when this enforcement system erodes at multiple levels. Mutations that inactivate tumour suppressors, hyperactivate oncogenes, or disable DNA repair reduce the cost of defection [4, 22, 23]. Chronic inflammation, scarring, or ageing remodel tissue architecture, creating permissive niches where deviant cells can hide. Immune escape mechanisms, from HLA downregulation to checkpoint ligand overexpression, disable extrinsic policing [16, 31]. From the contract viewpoint, this is not a single ‘breach’ but a progressive loss of enforcement capacity. Each lesion slightly shifts the balance of power between the organism and its cells. Eventually, the contract ceases to be credible: cells can defect and still prosper. At that point, clonal evolution accelerates, exploring new genotypes and phenotypes within the altered rules of the game [2, 22]. This reframing also clarifies why cancer is a generic risk of multicellularity. Any system built on cooperation among replicators must deal with cheating. In complex animals, the social contract between organism and cells is unusually intricate and therefore vulnerable at many points, including germ-line mutation, somatic mutation, environmental exposure, and age-related decline in surveillance [27, 32, 33].

## TUMOUR ECOLOGY

**Figure 2:**
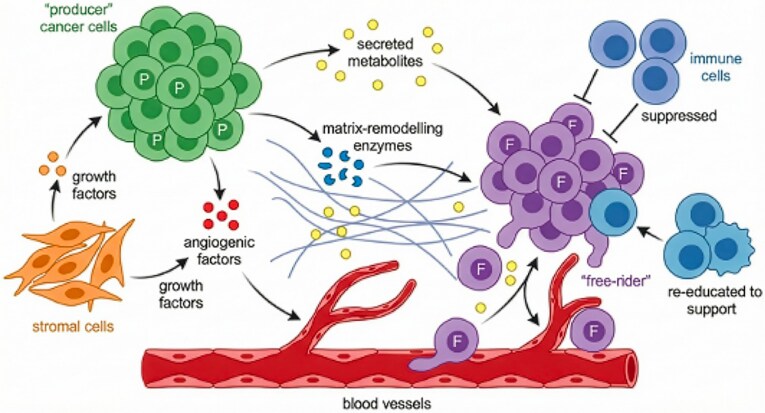
Public goods and free riding in tumour ecosystems. Tumour growth involves shared resources and niche modifications that can function as public goods. Some cancer cell subclones (‘producers’) invest in diffusible or structural factors such as VEGF, matrix-remodelling enzymes, or secreted metabolites that reshape the microenvironment. Other subclones (‘free riders’) exploit these goods without paying the metabolic costs. Producer and free-rider clones also interact with stromal and immune cells that can be co-opted into tumour-supporting programmes. The balance between production and exploitation can influence invasion, heterogeneity, metastasis, and therapy response [39, 41, 42].

Once the multicellular contract weakens, tumours do not simply become amorphous masses of selfish cells. They are structured, evolving populations that actively engineer their microenvironment: neoplastic cells secrete growth factors, cytokines, proteases, and extracellular matrix components; they induce angiogenesis, and they reprogram metabolism [17, 34–38]. Many of these products function as public goods. Diffusible factors or structural changes that benefit nearby cells regardless of who produced them [39, 40]. Fig. 2 depicts this tumour public-goods system, i.e. producer clones invest in public goods such as VEGF, matrix metalloproteinases, or lactate export, while free riders exploit these goods without paying the metabolic cost. In game-theoretic terms, subclones are the players. Secretion of shared factors (e.g. Vascular Endothelial Growth Factor (VEGF)) is cooperation, free-riding is defection, and payoffs are frequency- and space-dependent. The balance between producers and free riders can shape invasion fronts, metastatic potential, and therapy response [39, 41–44]. Supplementary Table 2 provides empirical examples of such cooperation and cheating: growth factor sharing, metabolic coupling between glycolytic and oxidative cells, and stromal co-option by cancer-associated fibroblasts [41, 45–47]. These patterns echo classical ecological scenarios in microbial communities [48] but are played out in a multicellular context. These experimental systems illustrate how growth factors, cytokines, amino-acid exchange, and lactate shuttling can act as public goods in cancer, enabling producer/free-rider dynamics and metabolic division of labour [41, 45–47, 49]. Like human societies, tumours often display hierarchical organization. Cancer stem-like cells may occupy privileged niches, resist therapy, and repopulate the tumour, while more differentiated progeny shoulder most of the proliferative burden [50, 51]. Subclones can occupy distinct spatial territories with unequal access to oxygen, nutrients, or vascular escape routes [1, 45, 52, 53]. In contract language, some clones become dominant ‘elites’, operationally, lineages with stem-like niche occupancy, spatial advantages in oxygen/vascular access, immune privilege, or measurable clonal dominance (e.g. high variant allele frequency or spatial prevalence), while others are confined to hypoxic or nutrient-poor zones. Because enforcement and resources vary across space and time, the payoff to any strategy (producer *vs* free rider, proliferative vs invasive) fluctuates. This instability can favour reversible switching between cooperative producer-like programmes (e.g. pro-angiogenic or matrix-remodelling states) and persister-like states that reduce costs and withstand stress, linking public-goods dynamics to non-genetic resistance. Phenotypic plasticity is therefore a natural evolutionary response to unstable enforcement regimes. Cells can switch between states, including transient persister-like or producer-like programmes, to match local conditions and evade policing or therapy [54, 55].

## PHENOTYPIC PLASTICITY AND THE EVOLUTION OF RESISTANCE

**Figure 3:**
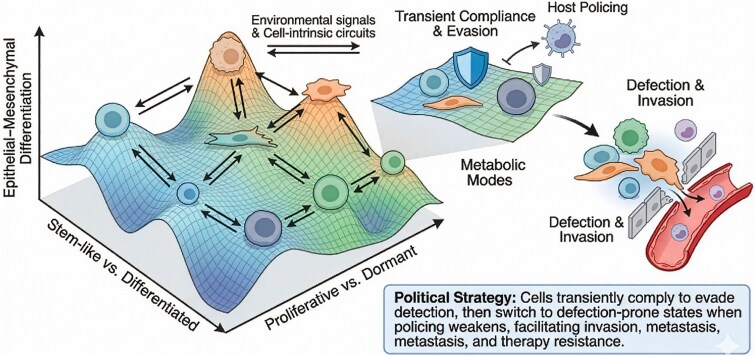
Phenotypic plasticity as a political strategy in somatic evolution. Cancer cells move on a multidimensional landscape of phenotypic states rather than occupying a single fixed identity. The schematic illustrates axes of epithelial-mesenchymal differentiation, stem-like versus differentiated programmes, proliferative versus dormant states, and metabolic modes. Environmental signals and cell-intrinsic circuits drive reversible transitions between states. From a multicellular social-contract perspective, plasticity functions as a political strategy: Cells can transiently comply with host constraints to evade detection, then switch to more defection-prone states when policing weakens, thereby facilitating invasion, metastasis, and therapy resistance [54, 55]. Therapy adds a third player to the game: Beyond the host and tumour, there is now an intelligent adversary that actively perturbs the system. Chemotherapy, radiotherapy, targeted agents, and immunotherapies impose strong, often abrupt selection pressures. Sensitive clones die; resistant ones survive and expand [1, 61].

**Figure 4:**
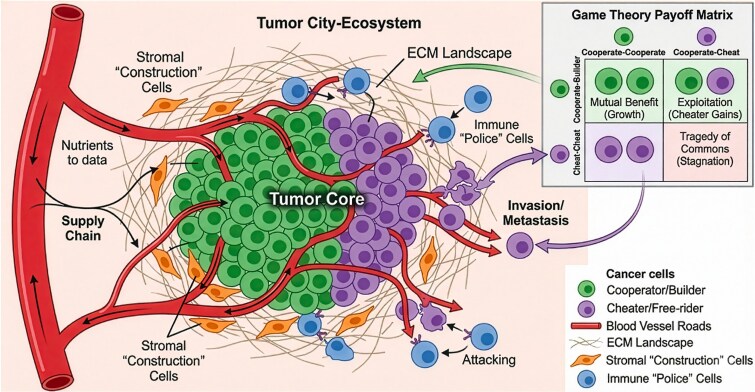
A three-way red queen race between host, tumour, and therapy.

In tumours where enforcement is uneven, and microenvironments fluctuate, reversible state switching can be strongly favoured. Phenotypic plasticity, reversible changes in cell state without permanent genetic alteration, is now recognized as a major contributor to metastasis and therapy resistance. Cells can shift between epithelial and mesenchymal phenotypes, between stem-like and differentiated states, or between proliferative and dormant programmes [54, 55]. Figure 3 illustrates this shapeshifting: cells move on a multidimensional landscape of adhesion, motility, metabolic mode, and stemness. When rules are unstable, because enforcement is failing, therapies are applied intermittently, or microenvironments fluctuate, cells that can rapidly change their behaviour have an advantage. The result is a Red Queen arms race: as we upgrade our treatments, tumours explore new resistance mechanisms, and hosts pay the cost in toxicity and organ damage [56, 57] (Fig. 4). Cancer evolution in treated patients involves three interacting players, i.e. host tissues, malignant cell populations, and therapeutic interventions. The diagram depicts reciprocal feedback loops: (i) host enforcement mechanisms (immune surveillance, tissue architecture, repair responses) constrain emerging clones; (ii) tumour lineages evolve genetic and phenotypic strategies to evade or subvert these constraints; and (iii) therapies impose additional selective pressures that reshape both host and tumour responses. This triadic interaction generates a Red Queen-like arms race in which each player must continuously adapt just to maintain its position. Therapies that ignore this three-way co-evolution risk selecting for highly adapted, treatment-refractory tumour states. Importantly, resistance is not only genetic. Plastic responses, quiescence, transition to drug-tolerant persister states, and niche-dependent survival can act as short-term truces in the conflict. They keep malignant lineages alive until more durable genetic solutions arise [55, 58–60].

## CLINICAL CONSEQUENCES: RENEGOTIATING THE CONTRACT

If we take the social-contract metaphor seriously, cancer therapy becomes not just a matter of killing malignant cells but of renegotiating the rules under which cells evolve in the body. Conventional protocols use maximum tolerated dose (MTD) regimens: push cells to the brink of viability in the hope of eradicating every clone. In evolutionary terms, MTD corresponds to an all-out war that aims to exterminate cheaters. But wars are risky: if any resistant clone survives, it inherits a largely empty habitat and can expand explosively [1, 62, 63]. Adaptive therapy offers a contrasting strategy. Instead of insisting that defectors must be eliminated, it aims to contain them within a manageable corridor, using sensitive clones as ecological competitors to suppress resistant ones [3, 61, 64]. Doses are modulated based on tumour burden, with treatment paused or reduced when sensitive populations decline too far. Supplementary Fig. 2 compares these approaches. Under MTD, resistant clones eventually dominate once sensitive ones are removed; under adaptive therapy, the physician acts as a contract manager, adjusting enforcement (drug pressure) to maintain a mixed population where resistant clones remain outcompeted. Beyond simple containment, evolutionary double binds try to force tumours into corners of the fitness landscape where every escape option is costly [64–66]. One drug may select for a phenotype that is particularly vulnerable to a second drug, or to immune attack, or to a metabolic bottleneck [42, 46, 47, 67]. Ecological traps extend this idea to the microenvironment, altering resource distributions, vascular patterns, or stromal interactions to lure malignant cells into states or locations where they are easier to control [68, 69, 70]. A social contract view naturally draws attention back to the host’s enforcement capacity. Strategies that maintain robust immune surveillance, preserve tissue architecture, and limit chronic inflammation support the original contract and reduce the opportunity space for cancer [27, 32, 33]. Preventive interventions, vaccination against oncogenic viruses, reduction of carcinogenic exposures, and management of metabolic and inflammatory states can be reinterpreted as investments in contract stability.

## CLINICAL TRANSLATION

The social-contract lens suggests that therapy can operate on more than one axis, i.e. not only reducing tumour burden, but also reshaping the selective conditions under which somatic evolution proceeds. Adaptive therapy and related optimal-control approaches can be interpreted as contract management. Dosing schedules are adjusted to maintain competitive suppression of resistant clones and avoid creating an ecological vacuum [3, 64, 71, 72]. This logic aligns naturally with Stackelberg game theory, where the physician acts as a leader who anticipates tumour responses and chooses interventions accordingly [73, 74]. Many tumour behaviours can be formalized as evolutionary games in which cancer cells are the players, cooperation and defection are strategies, and fitness payoffs emerge from interactions mediated by shared microenvironmental goods. For angiogenesis, VEGF-producing cells pay a cost to secrete a diffusible benefit that improves local perfusion, whereas non-producing cells can exploit that benefit, generating frequency-dependent selection between producers and free riders [39, 41, 75]. Depending on spatial structure and resource limitation, this interaction can favour either cooperation or defection and may even produce tragedy-of-the-commons dynamics, where short-term individual advantage degrades collective perfusion [76, 77]. These models suggest that therapy might sometimes shift the game being played by altering stromal context or resource maps, rather than only escalating cytotoxicity [78–80]. Ecological-trap concepts can be translated into concrete designs that manipulate movement and habitat choice. In glioma, the ‘cancer cell trap’ proposes attracting migrating cells into engineered sinks or constrained niches where they are easier to eradicate, turning dispersal into a liability [68, 69]. More generally, therapies that alter resource maps (oxygen, acidity, nutrients) or stromal interactions could steer malignant lineages into controllable regions of phenotype space. These clinical strategies connect to broader evolutionary framings. Advanced cancer has been described as resembling a speciation event, a transition to a quasi-independent lineage within the host, consistent with a multicellular contract that has become non-credible [81]. Closely related is the concept of cancer as ‘corruption’. Tumours exploit and co-opt signalling, enforcement, and coordination systems that normally stabilize cooperation, through deceptive signalling, collusion, and institutional capture [82]. Both framings sharpen the therapeutic question from ‘How do we kill cells?’ to ‘Which rules and enforcement mechanisms can we restore, substitute, or strategically exploit?’

## ANALOGY, LIMITS AND PROSPECTS

**Figure 5:**
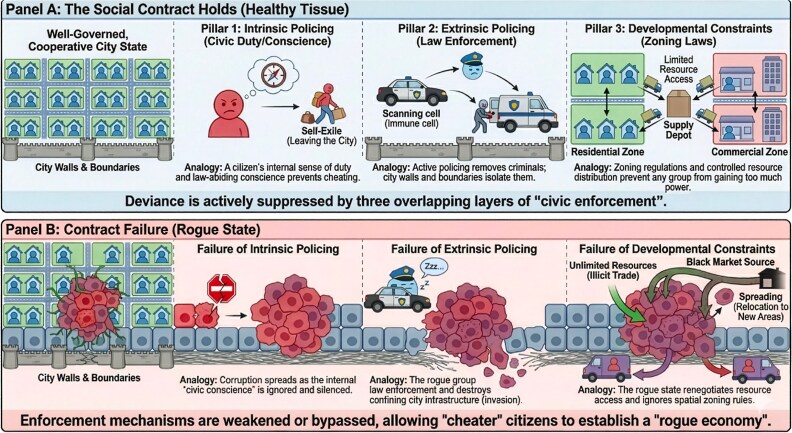
Civic analogy to illustrate the biological transition from cooperative tissue to malignancy. Panel a depicts healthy tissue maintained by three pillars of enforcement: Intrinsic policing (cell-cycle checkpoints and apoptosis), extrinsic policing (immune surveillance), and developmental constraints (architecture, niche limits, and resource distribution). Panel B depicts malignant progression as erosion of these pillars: Checkpoint defects, immune evasion, boundary breakdown, and vascular/metabolic rewiring that enable invasion and metastasis.

As shown in Fig. 5, stages of cancer development can be mapped onto phases of enforcement loss: early lesions where checkpoint failure or immune escape is episodically corrected; intermediate tumours where stromal co-option, angiogenesis, and spatial structure create locally self-sustaining niches; and advanced disease where boundaries are breached, and dissemination occurs. The civic language is deliberately provocative, but it keeps mechanistic referents in view, which constraints fail, how they fail, and how evolving lineages exploit the resulting opportunities [11, 83].

## CONCLUSION

There are obvious limits to the analogy introduced here. Cells have no intentions. Contracts are not literally negotiated. There is no conscious state. Still, collective-action failures in human societies highlight the same logic. In fact, cooperation without credible enforcement can unravel, e.g. climate change mitigation under the Paris Agreement depends on sustained compliance despite incentives to defect. The value of the analogy lies not in anthropomorphizing tumours but in focusing our attention on rules and enforcement. Which constraints matter most in each tissue, checkpoint pathways, niche geometry, immune patrols, metabolic bottlenecks? How do malignant lineages re-write these constraints as they evolve? Which therapeutic strategies strengthen the host’s bargaining position, and which inadvertently empower the tumour [84–90]? The social-contract framework suggests several testable questions. Can we build indices of contract robustness that integrate genomic integrity, immune competence, and tissue architecture, analogous to eco-evolutionary indices proposed for classifying tumours [27, 33, 83, 91]? How do specific public goods, niche modifications, and stromal alliances structure power relations within tumours, and how do these differ between early lesions and advanced metastases [40, 45, 83]? Can longitudinal sampling, single-cell omics, and spatial profiling follow how enforcement mechanisms and cheating strategies co-evolve during progression and therapy [1, 3, 51, 92]? And can we deliberately craft treatment sequences that maintain a residual multicellular order, preserving enforcement mechanisms where possible and exploiting the vulnerabilities of cheaters rather than merely escalating force [3, 61, 64]? Cancer is often framed as a purely genetic disease or a purely ecological one. Both views are indispensable, but they can obscure the fact that what is evolving is not just cell populations but also the rules under which they operate. Multicellular life rests on a fragile contract that keeps somatic evolution in check; cancer is the story of what happens when this contract fails, and new, rogue arrangements emerge. By bringing together clonal evolution, tumour ecology, phenotypic plasticity, and treatment strategies under the umbrella of a multicellular social contract, we gain a language for thinking about cancer as a political process inside the body: a struggle over resource allocation, policing, hierarchy, and resistance [83, 89, 90, 93, 94].

## Supplementary Material

Supplementary_Figures_eoag004

Supplementary_Tables_eoag004

## Data Availability

No new datasets were generated or analysed in this Review.
