# Supplementary material for: Cancer, collapse, and the politics of somatic evolution
Source: Evol Med Public Health. 2026 Jan 29;14(1):1–10. doi: 10.1093/emph/eoag004 (PMC13169519; doi:10.1093/emph/eoag004)
Supplement: Supplementary_Figures_eoag004 [file supplementary_figures_eoag004.docx]

**Supplementary Figure 1**


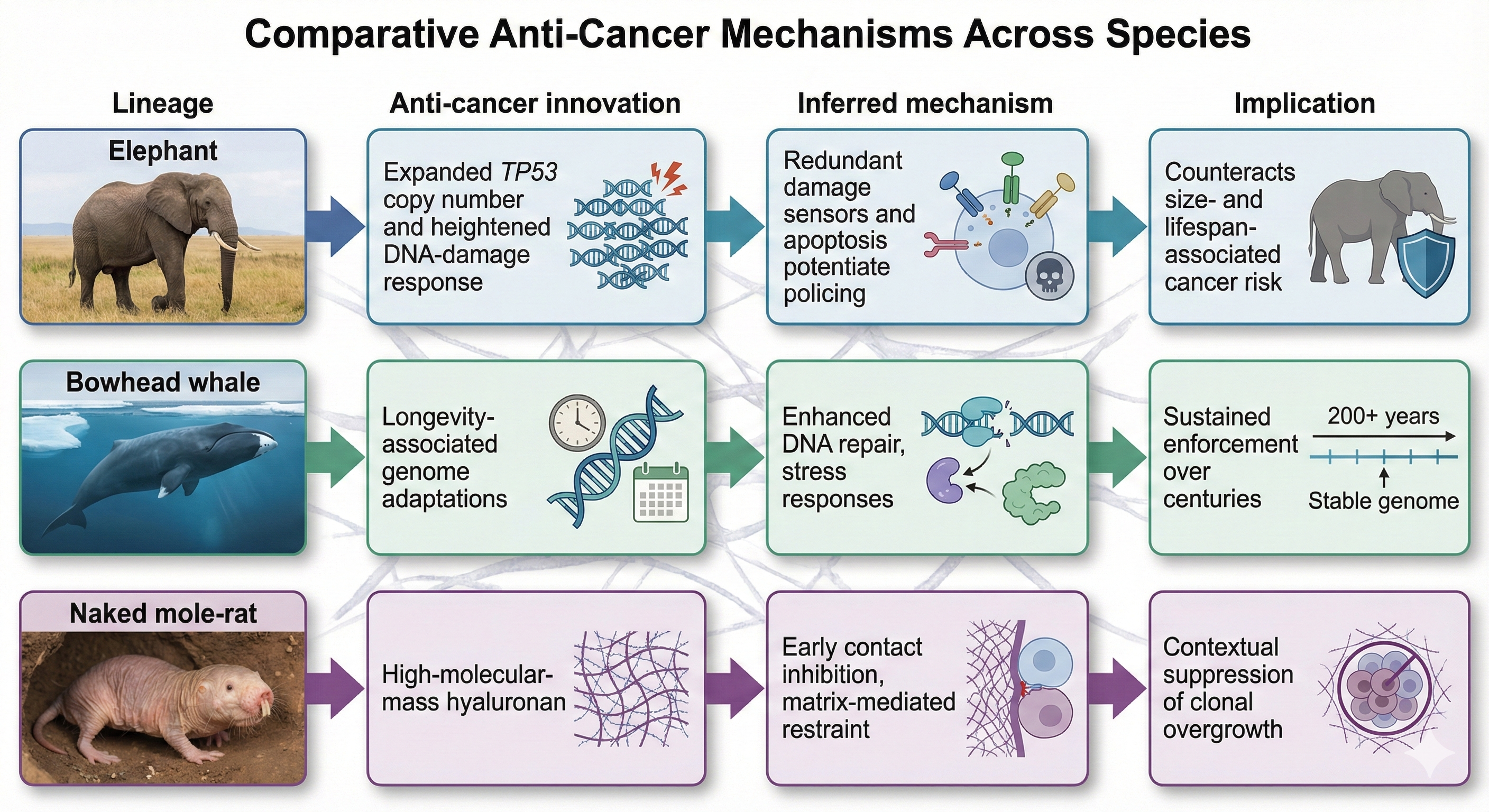


**Supplementary Figure 1:** Diverse evolutionary solutions to Peto’s Paradox. This schematic illustrates the distinct biological strategies evolved by three lineages to suppress cancer despite high cell counts or extended lifespans. The top row depicts the elephant’s reliance on TP53 copy number expansion to heighten DNA-damage sensitivity and trigger apoptosis (policing). The middle row highlights the bowhead whale’s adaptations for enhanced DNA repair, ensuring genomic stability over centuries. The bottom row demonstrates the naked mole-rat’s unique use of high-molecular-mass hyaluronan (HMM-HA) to enforce early contact inhibition, physically constraining clonal overgrowth via the extracellular matrix.

**Supplementary Figure 2**


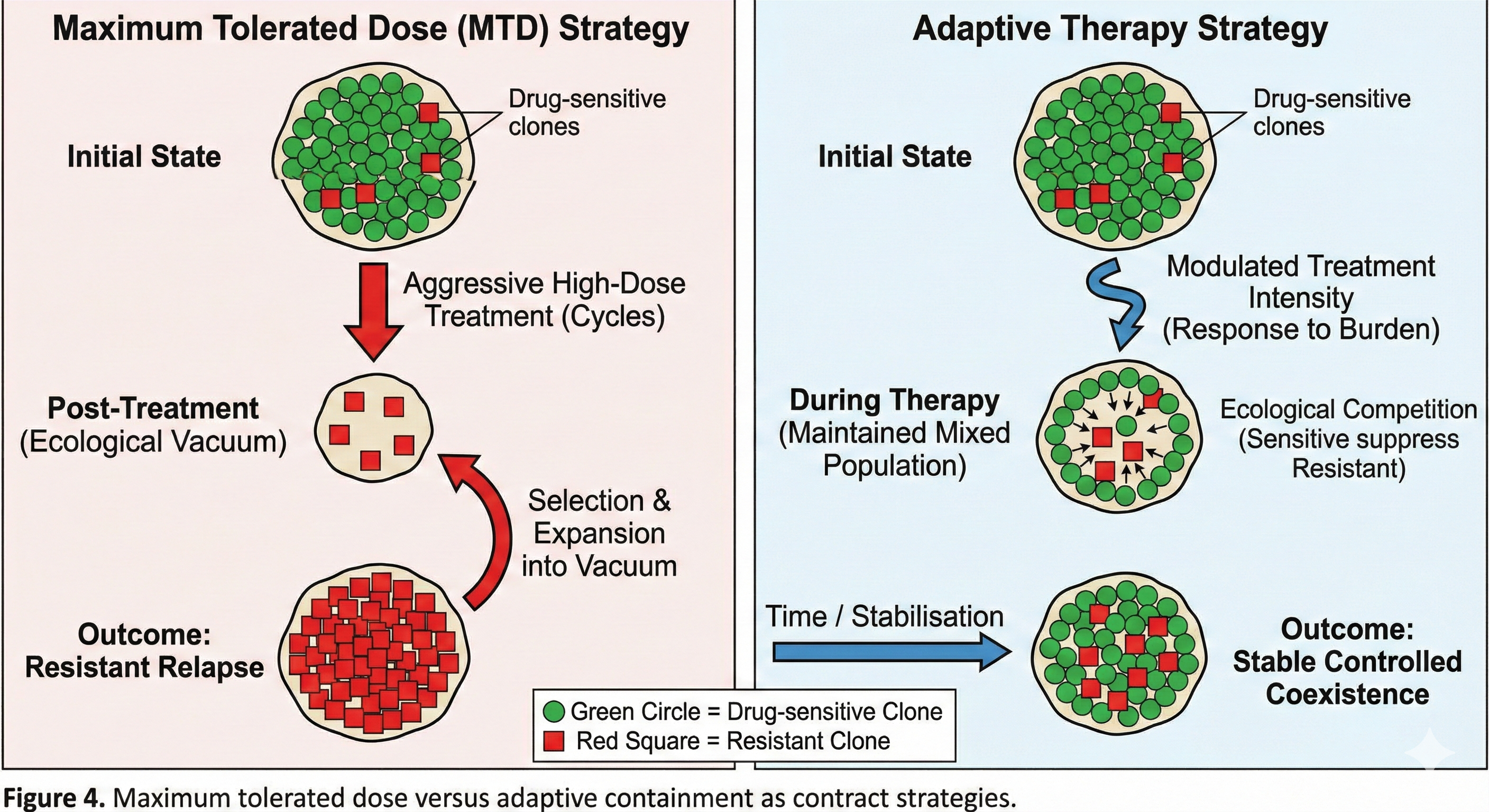


**Supplementary Figure 2**: Maximum tolerated dose versus adaptive containment as contract strategies. Conceptual comparison of two therapeutic logics. Under conventional maximum tolerated dose (MTD) strategies, repeated cycles of aggressive treatment eliminate most drug-sensitive clones but strongly select for resistant ones, which then expand into an ecological vacuum and drive relapse. Under adaptive therapy, treatment intensity is modulated in response to tumour burden to maintain a mixed population in which drug-sensitive clones suppress resistant ones via ecological competition. Rather than aiming for complete eradication of defectors, adaptive therapy seeks to contain them within a manageable corridor, effectively renegotiating the multicellular contract to stabilise a controlled coexistence (Gatenby et al., 2009; Zhang et al., 2017; Gatenby & Brown, 2020).
