# Supplementary material for: Cancer, collapse, and the politics of somatic evolution
Source: Evol Med Public Health. 2026 Jan 29;14(1):1–10. doi: 10.1093/emph/eoag004 (PMC13169519; doi:10.1093/emph/eoag004)
Supplement: Supplementary_Tables_eoag004 [file supplementary_tables_eoag004.docx]

**Supplementary Table 1:** Comparative DNA Repair Efficiency

| **Feature** | **Human (Baseline)** | **Bowhead Whale (Enhanced)** | **Impact on Longevity** |
| --- | --- | --- | --- |
| Primary Strategy | **"Policing"** (Apoptosis)    Damaged cells are triggered to commit suicide to prevent risk. | **"Maintenance"** (High-Fidelity Repair)    Damaged cells are repaired and retained to preserve tissue function. | Whale strategy prevents cell depletion, delaying aging. |
| DSB Repair Fidelity | **Moderate**    Non-Homologous End Joining (NHEJ) is often "error-prone," leaving small scars/deletions. | **High**    NHEJ repair is highly accurate with significantly fewer deletions or errors. | Whales accumulate fewer mutations per cell division despite larger body mass. |
| Repair Speed | **Standard**    DNA damage foci resolve over 24–48 hours. | **Rapid**    Double-Strand Breaks (DSBs) are resolved significantly faster than in humans. | Prevents genomic instability from cascading into cancer. |
| Key Protein | **p53** (Guardian)    Focuses on spotting damage and triggering arrest/death. | **CIRBP** (Cold-Inducible RNA-Binding Protein)    Expressed **~100x higher** than in humans; acts as a repair efficiency booster. | High CIRBP levels directly correlate with superior DNA repair capacity. |
| Inflammation | **High** (SASP)    Senescent cells secrete inflammatory signals, damaging neighbors. | **Attenuated**    Cells show reduced inflammatory secretions even when stressed. | Whales avoid "inflammaging"chronic inflammation associated with age. |

**Supplementary Table 2**: Cooperation, cheating and public goods in tumour ecosystems

| System | Cooperative trait | Ecological consequence |
| --- | --- | --- |
| Pancreatic neuroendocrine tumour | IGF-II production by a producer subclone with free-riding neighbours | Maintenance of mixed producer/non-producer populations via public-goods dynamics |
| Breast cancer xenograft | Minor subclones secrete IL-11 and VEGF-D | Microenvironmental remodelling, neutrophil recruitment, polyclonal metastasis |
| Pancreatic ductal adenocarcinoma | Autophagic alanine secretion by stellate cells feeds cancer TCA cycle | Cross-lineage metabolic coupling supports growth under hypoxia/nutrient stress |
| Hypoxic tumour regions | Lactate shuttling and utilisation | Division of labour in metabolism, survival under fluctuating oxygen |
